# Advances and challenges in oral health after a decade of the “Smiling Brazil” Program

**DOI:** 10.1590/S0034-8910.2015049005961

**Published:** 2016-01-14

**Authors:** Charleni Inês Scherer, Magda Duarte dos Anjos Scherer

**Affiliations:** I Programa de Pós-Graduação em Saúde Coletiva. Faculdade de Ciências da Saúde. Universidade de Brasília. Brasília, DF, Brasil; II Departamento de Saúde Coletiva. Faculdade de Ciências da Saúde. Universidade de Brasília. Brasília, DF, Brasil

**Keywords:** Dental Health Services, organization and administration, Public Health Dentistry, Manpower, Primary Health Care, Dentist’s Practice Patterns, Working Conditions, Public Health Policy, Review

## Abstract

**OBJECTIVE:**

To analyze oral health work changes in primary health care after Brazil’s National Oral Health Policy Guidelines were released.

**METHODS:**

A literature review was conducted on Medline, LILACS, Embase, SciELO, *Biblioteca Virtual em Saúde*, and The Cochrane Library databases, from 2000 to 2013, on elements to analyze work changes. The descriptors used included: primary health care, family health care, work, health care policy, oral health care services, dentistry, oral health, and Brazil. Thirty-two studies were selected and analyzed, with a predominance of qualitative studies from the Northeast region with workers, especially dentists, focusing on completeness and quality of care.

**RESULTS:**

Observed advances focused on educational and permanent education actions; on welcoming, bonding, and accountability. The main challenges were related to completeness; extension and improvement of care; integrated teamwork; working conditions; planning, monitoring, and evaluation of actions; stimulating people’s participation and social control; and intersectorial actions.

**CONCLUSIONS:**

Despite the new regulatory environment, there are very few changes in oral health work. Professionals tend to reproduce the dominant biomedical model. Continuing efforts will be required in work management, training, and permanent education fields. Among the possibilities are the increased engagement of managers and professionals in a process to understand work dynamics and training in the perspective of building significant changes for local realities.

## INTRODUCTION

Brazil has made advancements with its Unified Health System (SUS) by establishing universal and full care as its principles and by increasing the coverage of its Primary Health Care (PHC), through Family Health Care Strategy (FHCS). However, the biomedical health care model prevails, and it guides all professional practices, including dentistry.^4^ ^,^ ^13^ ^,^ ^35^ ^,^ ^48^ ^,^ ^50^ ^,^ ^54^ ^,^ ^55^

According to its last epidemiological survey, Brazil shifted its prevalence of caries from medium to low.^59^ Although results are nationally satisfactory, some factors call our attention: (a) regional differences in the prevalence and seriousness of caries are distinctive, which indicates a need for policies focused on equal care; (b) small reduction of caries in deciduous dentition (18.0%) and 80.0% of affected teeth remaining untreated; (c) significant deficit for older adults, despite adolescents’ and adults’ need for prosthetics having been decreased; and (d) prevalence of malocclusion requiring treatment in 10.0% of adolescents, which suggests a need for resizing the supply of dental procedures in secondary care.^32^ ^,^ ^33^ These results are associated with the profile of dental practice, characterized by the conduction of eminently clinical actions emphasizing restoring activities and preventive actions focusing on students, which were shown to be insufficient to meet the needs of the population.^31^

The Brazilian path to shift the direction of its oral health care model in PHC has found milestones that have a potential to drive work changes: (a) first *ConferênciaNacional de Saúde Bucal* (CNSB *–* National Oral Health Conference) in 1986, followed by the creation of Brazil’s National Oral Health Policy in 1989,^a^ and by the second CNSB in 1993;^9^ (b) inclusion of dental professionals in FHCS in 2000,^17^ facing the historical restriction of dealing with mother and their children and established a federal financial incentive; creation of new national syllabus guidelines for undergraduate courses in the health care field;^3^ and approval of rules and guidelines to include oral health care teams in FHCS in 2001; (c) release of the *Programa Brasil Sorridente* (“Smiling Brazil” Program) in 2004^23^ and the third CNSB, which contributed to democratic and forward-looking production on the topic; (d) common and specific responsibilities of oral health care professionals in Brazil’s National Basic Health Care Policy in 2006,^b^ which were restated in 2011.^c^

**Figure 1:**
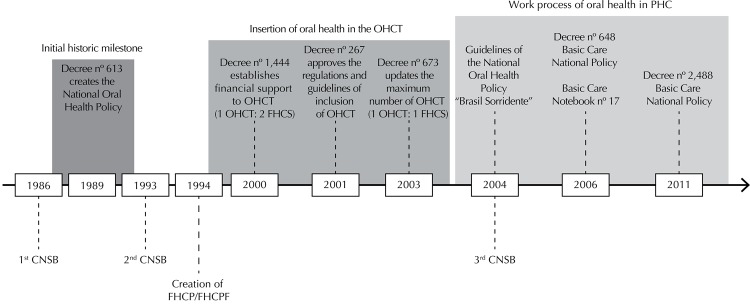
Timeline identifying the moments and regulations that mark the incentive to changes in the oral health work process in Primary Health Care in Brazil.

CNSB: *Conferência Nacional de Saúde Bucal* (National Oral Health Conference); FHCP: Family Health Care Program; FHCS: Family Health Care Strategy; OHCT: Oral Health Care Team

The recent path of oral health care signals that a new model is being built in the country (Figure 1).

The “Smiling Brazil” Program, as a guideline of Brazil’s National Oral Health Policy (PNSB),^d^ is the largest public oral health care program in the world and it has turned a decade old in 2014.^28^ Changes were made to the work of oral health care teams in PHC over that period, in a way to meet the goals for readjusting the health care model.^20^

The PHC is a potential space for innovation in the management and organization of the work process, one of the central axes for rearranging SUS’s health care.

This article intended to analyze the oral health work changes in primary health care after Brazil’s National Oral Health Policy Guidelines were released.

## METHODS

The literature review was guided by analyzing elements of work changes in oral health in PHC, according to regulations in effect,^27^ ^,^ ^c^ ^,^ ^d^ and from the publication of the Guidelines of PNSB. The following elements were used to analyze the changes in oral health in PHC: welcoming, bonding, and accountability; extension and improvement of care; intersectorial actions; educational actions; permanent education; fostering of popular participation and social control; completeness; planning, monitoring, and evaluation of actions; integrated teamwork; and working conditions. The guiding questions were: “Which analyzing elements are mentioned?”, “Are reports of work changes, advances, or difficulties present?”, “Are recommendations, complaints, or suggestions for oral health work in PHC present?”.

Studies were collected in Medline (via PubMed), LILACS, Embase, SciELO, *Biblioteca Virtual em Saúde* (BVS), and The Cochrane Library (via Bireme) databases. Publications from 2000 to 2013 were included (until November 3, 2013). A period prior to 2004 was included, when the Guidelines of PNSB were released, after experiences and agreements among the several players involved in the discussion and that may have been research topics in published scientific journals.

The descriptors used in the search on PubMed were: (“primary health care” OR “family health” OR “work” OR “health policy”) AND (“dental health services” OR “dentistry” OR “oral health”) AND (“Brazil”). The descriptors were used in English and Portuguese in the remaining databases. The combination was conducted with the use of boolean operators “AND” and “OR”, as well as terms from the Medical Subject Headings (MeSH) or analogous ones available in each surveyed database.

Data were collected by one of the authors, whereas the other two, who were familiar with the topic and the method, undertook the selection and evaluation of studies. A total of 77 articles were found on Medline, 538 on LILACS, four on Embase, 67 on SciELO, 338 on BVS, and 13 on The Cochrane Library, which totaled 1,037 articles.

We included the articles that concerned the work, practices, or change of model in oral health in PHC, selected by title. The selected articles were those that discussed the organization and management of work processes in oral health, especially about teamwork in PHC, a central element of the rearrangement of SUS’ health care. Studies of theoretical reflection, essays, and theses were excluded.

**Figure 2:**
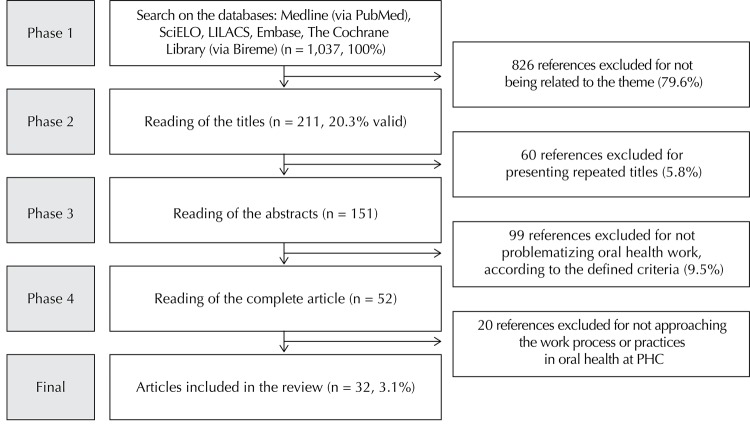
Search, selection, inclusion, and exclusion of studies on the work process in oral health in Primary Health Care.

We selected 211 articles. Sixty of them were excluded for being repeated in the bases, which resulted in 151 studies whose abstracts should be read and 52 to be fully read. At the end, 32 articles were included in the final analysis (Figure 2).

PHC: Primary Health Care

General publication aspects, methodological characteristics, and main results were identified and analyzed based on their elements (Table 1). Information screening was independently conducted by the researchers and compared in a meeting for consensus. Agreed items were considered proper and included in the description of results. These were grouped according to their previous topic categories (elements for analysis) and analyzed in a descriptive manner.

**Table 1:** General aspects of selected studies, methodological characteristics, and study subjects.

Year	Location	Author	Journal	Title	Study type and methodology		Study subjects
2013	SP	Pezzato LM, L’Abbate S, and Botazzo C	Ciência & Saúde Coletiva, 18(7):2095-104	*A produção de micropolíticas no processo de trabalho em saúde bucal: uma abordagem sócio-analítica* (The production of micro-policies in the work process in oral health care: a socioanalytical approach)	Qualitative	Socioanalytical approach	Workers (OHCT) and users
2013	MG	Sanglard-Oliveira CA, Werneck MAF, Lucas SD, and Abreu MHNG	Ciência & Saúde Coletiva, 18(8):2453-60	*Atribuições dos Técnicos em Saúde Bucal na Estratégia Saúde da Família em Minas Gerais, Brasil* (Attributions of oral health technicians in the Family health care strategy in Minas Gerais, Brazil)	Qualitative	Cross-sectional, descriptive study Telephone interview	Workers (DS)
2013	PI	Moura MS, et al	Ciência & Saúde Coletiva, 18(2):471-80	*Saúde bucal na estratégia de saúde da família em um colegiado gestor regional do estado do Piauí* (Oral Health in the strategy of family health care in a managing regional collegiate of Piauí state)	Qualitative	Cross-sectional study Questionnaire	Workers (DS)
2012	ES	Esposti CDD, Oliveira AE, Santos Neto ET, and Zandonade E	Saúde Soc. São Paulo, 21(2):372-85	*O processo de trabalho do técnico em saúde bucal e suas relações com a equipe de saúde bucal na Região Metropolitana da Grande Vitória, Espírito Santo, Brasil* (The work process of oral health care technicians and their relationships with oral health care teams in the metropolitan region of Grande Vitória, Espírito Santo, Brazil)	Qualitative	Semi-structured interview	Workers (DS)
2012	PE	Pimentel FC, et al	Cad. Saúde Pública, 28 Sup:S146-S157	*Caracterização do processo de trabalho das equipes de saúde bucal em municípios do Estado de Pernambuco, Brasil, de acordo com o tamanho da população: a partir de links da comunidade para a organização dos cuidados clínicos.* (Characterization of the work process of oral health care teams in municipalities in the state of Pernambuco, Brazil, according to the population size: from community links for the organization of clinical care).	Qualitative	Structured questionnaire	Workers (OHCT)
2011	SP	Cunha BAT, Marques RAA, Castro CGJ, and Narvai PC	Saúde Soc, 20(4):1033-45	*Saúde bucal em Diadema: da odontologia escolar à estratégia saúde da família* (Oral health in Diadema: from school dentistry to the family health care strategy)	Qualitative	Semi-structured interview and document analysis	Managers and worker (Mayors, MHCO, OHC, and CD)
2011	SP	Mialhe FL, Lefèvre F, Lefèvre AMC	Ciência & Saúde Coletiva, 16(11):4425-32	*O agente comunitário de saúde e suas práticas educativas em saúde bucal: uma avaliação qualiquantitativa* (Community health care agents and their oral health-related educational practices: a qualitative and quantitative evaluation)	Qualitative and quantitative	Semi-structured interview	Workers (CHCA)
2011	PE	Silva SF et al	Ciência & Saúde Coletiva, 16(1):211-20	*Análise do avanço das equipes de saúde bucal inseridas na Estratégia Saúde da Família em Pernambuco, região Nordeste, Brasil, 2002 a 2005* (Analysis of the advancement of oral health care teams in the family health care strategy in Pernambuco, Northeast region, Brazil, 2002 to 2005)	Quantitative	Descriptive exploratory study	Secondary data (SIA-SUS)
2011	BA	Rodrigues AAAO, Nascimento MAA, Fonsêca GS, and Siqueira DVS	Rev. Baiana Saúde Pública, 35(3):695-709	*Saúde bucal na estratégia saúde da família em um município do semiárido baiano* (Oral health in the family health care strategy in a municipality in Bahia’s semi-arid zone)	Qualitative	Semi-structured interview and observation	Workers (FHCT and OHCT)
2010	PE	Pimentel FC, et al	Ciência & Saúde Coletiva, 15(4):2189-96	*Análise da atenção à saúde bucal na Estratégia de Saúde da Família do Distrito Sanitário VI, Recife - PE* (Analysis of oral health care in the Family Health Care Strategy in Sanitary District VI, Recife - PE)	Qualitative	Descriptive study Semi-structured interview and secondary data	Managers and workers (OHC, DS, and nurse)
2010	BA	Rodrigues AAAO, Santos AM, and Assis MMA	Ciência & Saúde Coletiva, 15(3):907-15	*Agente comunitário de saúde: sujeito da prática em saúde bucal em Alagoinhas, Bahia* (Community health care agent: subject of the oral health care practice in Alagoinhas, Bahia)	Qualitative	Semi-structured interview, observation, and document analysis	Workers (CHCA and OHCT)
2010	CE	Nuto SAS, Oliveira GC, Andrade, JV, and Maia MCG	Rev. APS, 13(4):505-9	*O acolhimento em saúde bucal na estratégia de saúde da família, Fortaleza-CE: um relato de experiência* (Welcoming in oral health in the family health care strategy, Fortaleza-CE: an account of an experiment)	Qualitative	Experiment report	Workers (OHCT and CHCA), users, and scholars
2010	PI	Moura MS, et al	Ciência & Saúde Coletiva, 15(supl.1): 1487-95	*Perfil e práticas de saúde bucal do agente comunitário de saúde em municípios piauienses de pequeno porte* (Oral health profile and practices of community health care agents in small-sized municipalities in Piaúi)	Qualitative	Cross-sectional, observational, descriptive study Questionnaire	Workers (CHCA)
2010	PE	Martelli PJL, et al	Ciência & Saúde Coletiva, 15(Supl. 2):3243-8	*Perfil do cirurgião dentista inserido na Estratégia de Saúde da Família em municípios do estado de Pernambuco, Brasil* (Profile of dentist surgeons in the Family Health Care Strategy in municipalities in Pernambuco state, Brazil)	Qualitative	Descriptive, exploratory, and cross-sectional case study Questionnaire	Workers (DS)
2010	SC	Faccin D, Sebold R, and Carcereri DL	Ciência & Saúde Coletiva, 15(Supl. 1):1643-52	*Processo de trabalho em saúde bucal: em busca de diferentes olhares para compreender e transformar a realidade* (Work process in oral health care: searching for different opinions in order to understand and transform reality)	Qualitative	Semi-structured interview	Workers (FHCT and OHCT)
2009	MG	Lourenço EC, Silva ACB, Meneghin MC, and Pereira AC	Ciência & Saúde Coletiva, 14(Supl. 1):1367-77	*A inserção dos serviços de saúde bucal no Programa Saúde da Família no Estado de Minas Gerais* (The inclusion of oral health care services in the family health care program in Minas Gerais state)	Qualitative and quantitative	Questionnaires	Workers (FHCT and OHCT)
2009	PR/SP	Nascimento AC, Moysés ST, Bisinelli JC, and Moysés SJ	Rev. Saúde Pública, 43(3):455-62	Oral health in the family health strategy: a change of practices or semantics diversionism	Qualitative	Focal Group	Workers (DS)
2009	BA	Rodrigues AAAO, Gallotti AP, Pena SFA, and Ledo CAS	Rev. Baiana Saúde Pública, 33(4):582-94	*Saúde bucal no programa de saúde da família na cidade de Feira de Santana (BA): o perfil do cirurgião-dentista* (Oral health in the family health care program in the city of Feira de Santana - BA - the profile of a dentist surgeon)	Quantitative	Questionnaire	Workers (DS)
2009	RN	Rocha ECA and Araújo MAD	Rev. Adm. Pública, 43(2): 481-517	*Condições de trabalho das equipes de saúde bucal no Programa Saúde da Família: o caso do Distrito Sanitário Norte em Natal, RN* (Working conditions of oral health care teams in the family health care program: the case of North Sanitary District in Natal, RN)	Qualitative and quantitative	Descriptive exploratory study Questionnaire	Workers (DS)
2009	RN	Holanda ALF, Barbosa AAA, and Brito EWG	Ciência & Saúde Coletiva, 14(Supl. 1):1507-12	*Reflexões acerca da atuação do agente comunitário de saúde nas ações de saúde bucal* (Reflections regarding the performance of community health care agents in oral health actions)	Qualitative	Experiment report	Workers (DS)
2008	PR	Koyashiki GAK, Alves-Souza RA, and Garanhani ML	Ciência & Saúde Coletiva, 13(4):1343-54	*O trabalho em saúde bucal do Agente Comunitário de Saúde em Unidades de Saúde da Família* (The oral health work of Community Health care Agents in Family Health Care Units)	Qualitative	Interview	Workers (CHCA)
2008	BA	Dos Santos AM, Assis MMA, Nascimento, MAA, and Jorge, MSB	Rev. Saúde Pública 2008;42(3):464-70	*Vínculo e autonomia na prática de saúde bucal no Programa Saúde da Família* (Bonding and autonomy in the oral health practice in the family health care program)	Qualitative	Critical-reflexive approach Semi-structured interview and observation	Workers (FHCT and OHCT) and users
2008	BA	Chaves SCL and Vieira-da-Silva LM	Health Policy 86(1):119-28	Inequalities in oral health practices and social space: an exploratory qualitative study	Qualitative	Exploratory study Semi-structured interview	Workers and managers (DS and OHC) and users
2008	PE	Martelli PJL, et al	Ciência & Saúde Coletiva, 13(5):1669-74	*Análise do modelo de atenção à saúde bucal em municípios do estado de Pernambuco* (Analysis of the oral health care model in municipalities in Pernambuco state)	Qualitative	Semi-structured interview	Managers (OHC)
2008	PE	Pimentel FC, et al	Rev. Baiana Saúde Pública, 32(2): 253-64	*Evolução da assistência em saúde bucal na estratégia de saúde da família do município do Recife (PE) no período de 2001 a 2007* (Evolution of the oral health care in the family health care strategy in the municipality of Recife - PE - from 2001 to 2007)	Quantitative	Descriptive study	Secondary data (SIA-SUS)
2008	RN	Almeida GC and Ferreira MA	Cad. Saúde Pública, 24(9):2131-40	*Saúde bucal no contexto do Programa de Saúde da Família: práticas de prevenção orientadas a saúde individual e pública* (Oral health in the context of the Family Health Care Program: prevention practices focusing on individual and public health)	Qualitative and quantitative	Descriptive exploratory study Structured interview Document analysis	Workers (DS) Secondary data (SIAB and SIA-SUS)
2008	PA	Emmi DT and Barroso RFF	Ciência & Saúde Coletiva, 13(1):35-41	*Avaliação das ações de saúde bucal no Programa Saúde da Família no distrito de Mosqueiro, Pará* (Evaluation of oral health actions in the family health care program in the district of Mosqueiro, Pará state)	Qualitative	Descriptive study Questionnaire	Users
2007	BA	Dos Santos AM, et al	Cad. Saúde Pública, 23(1):75-85	*Linhas de tensões no processo de acolhimento das equipes de saúde bucal do Programa Saúde da Família: o caso de Alagoinhas, Bahia, Brasil* (Tension lines in the process of receiving oral health care teams of the family health care program: the case of Alagoinhas, Bahia, Brazil)	Qualitative	Semi-structured interview and observation	Workers (FHCT and OHCT) and users
2007	BA	Chaves SCL and Silva LMV	Ciência & Saúde Coletiva, 12(6):1697-710	*As práticas profissionais no campo público de atenção à saúde bucal: o caso de dois municípios da Bahia* (Professional practices in the public oral health care: the case of two municipalities in Bahia)	Qualitative	Semi-structured interview	Workers (DS)
2007	RN	Souza TM and Roncalli AG	Cad. Saúde Pública, 23(11):2727-39	*Saúde bucal no Programa Saúde da Família: uma avaliação do modelo assistencial* (Oral health in the family health care program: an evaluation of the care model)	Qualitative	Structured interview, observation, and document analysis	Managers (OHC or person in charge) and workers (DS)
2005	PR	Baldani MH, Fadel CB, Possamai T, and Queiroz MGS	Cad. Saúde Pública, 21(4):1026-35	*Inclusão de serviços de saúde bucal no Programa Saúde da Família no Estado do Paraná* (Inclusion of oral health care services in the family health care program in Paraná State)	Qualitative and quantitative	Questionnaires	Managers (OHC or MHCO) and workers (DS)
2005	BA	Rodrigues AAAO and Assis MMA	Rev. Baiana Saúde Pública, 29(2):273-85	*Oferta e demanda na atenção à saúde bucal: o processo de trabalho no Programa Saúde da Família em Alagoinhas – Bahia* (Supply and demand in oral health care: the work process of the family health care program in Alagoinhas - Bahia)	Qualitative	Semi-structured interview, observation, and document analysis	Managers (Mayor, MHCO, and OHC) and workers (FHCT and OHCT + CHCA)

CHCA: community health care agent; DS: Dental Surgeon; OHC: Oral Health Coordinator; OHCT: Oral Health Care Team; FHCT: Family Health Care Team; SIA-SUS: *Sistema de Informações Ambulatoriais* (System of Ambulatorial Information) of SUS; SIAB: *Sistema de Informação de Atenção Básica* (Basic Health Care Information System); MHCO: Municipal Health Care Officer

## RESULTS

Most studies were from the Northeast (n = 21; 65.6%), Southeast (n = 6; 18.7%) and South regions (n = 3; 9.3%) (Table 1). We identified qualitative (n = 24; 75.0%) and quantitative studies (n = 3; 9.3%). A combination of quantitative and qualitative methods was used in 15.6% of the articles (Table 2). In general, the main subjects were FHCT workers, dental surgeons (DS) being highlighted. Around a third of them included managers and users (Table 1). All studies (n = 32) mentioned one or more work-analyzing elements, but none included all of them (Table 2).

**Table 2:** Selected studies on the analyzing elements of oral health work in Primary Health Care in Brazil pointing out challenges and changes.

Region	State	Year	Author	Analyzing elements in the studies	

				Indicating challenges	Pointing out changes
South	PR	2005	Baldani MH, Fadel CB, Possamai T, and Queiroz MGS	1, 7, 9, and 10	4
	PR	2008	Koyashiki GAK, Alves-Souza RA, and Garanhani ML	5 and 9	7
	SC	2010	Faccin D, Sebold R, and Carcereri DL	1, 3, 7, 9, and 10	2
Southeast	MG	2009	Lourenço EC, Silva ACB, Meneghin MC, and Pereira AC	5, 8, 9, and 10	1
	SP	2010	Cunha BAT, Marques RAA, Castro CGJ, and Narvai PC	1 and 7	4 and 5
	SP	2011	Mialhe FL, Lefèvre F, Lefèvre AMC	2 and 5	0
	ES	2012	Esposti CDD, Oliveira AE, Santos Neto ET, and Zandonade E	9	5 and 10
	SP	2013	Pezzato LM, L’Abbate S, and Botazzo C	6	1 and 7
	MG	2013	Sanglard-Oliveira CA, Werneck MAF, Lucas SD, and Abreu MHNG	0	2, 4, and 7
South and Southeast	PR	2009	Nascimento AC, Moysés ST, Bisinelli JC, and Moysés SJ	1, 9	4, 7
	SP			1	0
Northeast	BA	2005	Rodrigues AAAO and Assis MMA	7 and 9	0
	BA	2007	Santos AM, Assis MMA, Rodrigues AAAO, Nascimento MAA, Jorge MSB	4	0
	BA	2007	Chaves SCL and Silva LMV	7	0
	RN	2007	Souza TM and Roncalli AG	1, 8, 9, and 10	3
	BA	2008	Santos AM, Assis MMA, Nascimento MAA, and Jorge MSB	1 and 10	4
	BA	2008	Chaves SCL and Vieira-da-Silva LM	2 and 7	0
	PE	2008	Martelli PJL, et al	1, 5, 8, and 10	9
	PE	2008	Pimentel FC, et al	7 and 8	1
	RN	2008	Almeida GC and Ferreira MA	1 and 8	2
	BA	2009	Rodrigues AAAO, Gallotti AP, Pena SFA, and Ledo CAS	10	2 and 5
	RN	2009	Rocha ECA and Araújo MAD	1 and 10	0
	RN	2009	Holanda ALF, Barbosa AAA, and Brito EWG	7 and 9	5
	PE	2010	Pimentel FC, et al	1, 6, 7, 8, 9	2
	BA	2010	Rodrigues AAAO, Santos AM, and Assis MMA	9	7
	CE	2010	Nuto SAS, Oliveira GC, Andrade JV, and Maia MCG	1	4 and 5
	PI	2010	Moura MS, et al	7	5
	PE	2010	Martelli PJL, et al	1, 5, and 10	2
	PE	2011	Silva SF, et al	7	1 and 5
	BA	2011	Rodrigues AAAO, Nascimento MAA, Fonsêca GS, and Siqueira DVS	5, 7, 8, and 10	0
	PE	2012	Pimentel FC, et al	1, 5, 6, 7, and 8	9
	PI	2013	Moura MS, et al	5, 7, 9, and 10	0
North	PA	2008	Emmi DT and Barroso RFF	0	1 and 2

1: extension and improvement of care; 2: educational actions; 3: intersectorial actions; 4: welcoming, bonding, and accountability; 5: permanent education in health; 6: fostering of popular participation and social control; 7: completeness; 8: planning, monitoring, and evaluation of actions; 9: integrated teamwork; 10: working conditions; 0: Absence of analyzing elements in the study; States: PR: Parana; SC: Santa Catarina; MG: Minas Gerais; SP: Sao Paulo; ES: Espirito Santo; BA: Bahia; PE: Pernambuco; RN: Rio Grande do Norte; PI: Piaui; PA: Para

The few advances in oral health work focused on educational actions; permanent education actions; welcoming, bonding, and accountability. The main challenges related to completeness; extension and improvement of care; teamwork; planning, monitoring, and evaluation of actions; and working conditions. Few studies^14^ ^,^ ^36^ ^,^ ^38^ ^,^ ^39^ ^,^ ^57^ included fostering of popular participation and social control and intersectorial actions.

Among the 32 studies analyzed, 19 of them mentioned the topic of completeness. Fourteen of those^5^ ^,^ ^7^ ^,^ ^8^ ^,^ ^10^ ^,^ ^14^ ^,^ ^16^,^29^ ^,^ ^30^ ^,^ ^37^ ^,^ ^38^ ^,^ ^41^ ^,^ ^44^ ^,^ ^53^ pointed out difficulties to restructure oral health in PHC, overcome the practices in the traditional school dentistry model and create new possibilities, such as the family approach and diagnosis of the health care situation.^1^ ^,^ ^10^ ^,^ ^14^ ^,^ ^34^ ^,^ ^39^ Actions focusing on clinical care and excess emphasis on technique and specialty persisted, and traditional preventive and educational practices prevailed.^7^ ^,^ ^14^ ^,^ ^41^ The oral health care teams found difficulties in practices related to FHCT, such as house calls by dentists, actions to prevent illnesses and promote health, as well as meetings and actions for articulation with the community.^5^ ^,^ ^39^

Insufficient changes related to completeness were presented in five studies^18^ ^,^ ^32^ ^,^ ^36^ ^,^ ^43^ ^,^ ^45^ (26.3%), which reported the introduction of care focused on the user, with a space for dialog and for the bringing together knowledge encompassing oral health. Unlike individual actions, the group actions and the advances in the preventive view and the practice of health education of professionals were expressive in FHCT.^1^ ^,^ ^14^ ^,^ ^38^ Another change regards to the oral health technicians, who spent more of their time in preventive and collective activities than in care activities.^45^

Two studies mentioned teamwork and showed that most DS reported integration with their teams, but only a few took part in meetings or used single records.^5^ ^,^ ^19^ The work of a DS was rarely inserted in shared practices with professionals of other fields, as their actions were autonomously, independently, and individually developed.^39^

We observed obstacles for teamwork also among dental professionals.^13^ The DS recognized their relationship with oral health technicians was damaged by the lack of information on the work process, due to being uncertain of how liable they were regarding the activities of technicians, and also due to being afraid of technicians becoming practical dentists and taking their space in the job market. On the other hand, the DS appreciated the participation of technicians in the reorganization of dental work and in the construction of a relationship of partnership and cooperation.

Intersectorial actions were mentioned in two of the 32 studies,^14^ ^,^ ^57^ related to oral health prevention and education actions developed in the community or at schools.^57^ We observed intersectorial actions to be volunteer practices in some of the teams, not reaching the expected impacts. Such circumstance may lead professionals to disbelief regarding FHCT, considering the inability of the health sector to deal with social determining factors of the health-disease process in an isolated manner.

Educational actions were present in nine studies,^1^ ^,^ ^8^ ^,^ ^11^ ^,^ ^14^ ^,^ ^22^ ^,^ ^26^ ^,^ ^38^ ^,^ ^42^ ^,^ ^45^ of which seven pointed out advances in the practices of professionals concerning what is recommended by the guidelines of PNSB. According to Martelli et al,^22^ 92.3% of the DS considered them relevant. Among those, 89.6% reported conducting them. Rodrigues et al^42^ identified that all DS conducted health education activities, at their health care units or schools, nursing homes, churches, and daycare facilities. The actions that were most reported were the ones of prevention and promotion in groups,^1^ ^,^ ^14^ highlighting oral health technicians as the ones responsible for them.^45^ The ones of education in health were more present in the daily lives of FHCT professionals.^38^ ^,^ ^42^

According to Mialhe et al,^26^ the educational activities in oral health were conducted sporadically and mainly focused on pregnant women, mothers, and their children, in a vertical model of transmission of information, targeting changes in individual behaviors and incorporation of healthy habits. That vision was shared by the population, which considered oral hygiene instructions as one of the most important improvements.^11^

Eight studies^1^ ^,^ ^19^ ^,^ ^21^ ^,^ ^37^ ^-^ ^44^ ^,^ ^57^ reported that planning, monitoring, and evaluation of actions were insufficient practices, and indicated difficulties in the conduction of surveys to recognize population needs, considering social and epidemiological characteristics.^38^ ^,^ ^44^ Despite the advances, oral health needs much investment, besides the control and evaluation of its actions through information systems, which strengthen planning and decision-making.^37^

Fostering of popular participation and social control was mentioned in three studies^36^ ^,^ ^38^ ^,^ ^39^ as an action to be stimulated by teams. Pezatto et al^36^ pointed out that the appropriation of oral health topics by social control spaces is one of the challenges in implementing oral health care services in SUS.

The extension and improvement of oral health care were mentioned in 19 of the 32 studies. Among those, five^11^ ^,^ ^19^ ^,^ ^36^ ^,^ ^37^ ^,^ ^53^ mentioned advances, but difficulties prevailed (73.6%) regarding meeting the needs of the related population.^5^ Excessive demand was highlighted as a negative aspect, with predominance of healing actions by the DS.^19^ Even with the extended access to oral health care services, organizing the demand was a critical bottleneck, as there are several gateways, large repressed demand, and little supply.^19^ ^,^ ^32^ ^,^ ^34^ Increasing and improving care requires facing challenges related to insufficient public investments; to the difficulties in referring patients to specialty services; the actions focusing on clinical care with excess emphasis on technique and specialty; and the rising demand for services by the population, focused on healing actions.^34^ Despite the difficulties, we observed positive aspects in the studies: limiting of clients, enabling better supervision; changes in the profile of dental procedures conducted; and population-based coverage according to the minimum limit as per the Ministry of Health.^11^ ^,^ ^37^ ^,^ ^53^

Establishing welcoming, bonding, and accountability allows negotiating with users and professionals of full health care, which helps the therapeutic act to be focused on the professional, however being conducted according to the user’s wishes. These elements were mentioned by seven studies.^5^ ^,^ ^10^ ^,^ ^32^ ^,^ ^34^ ^,^ ^45^ ^-^ ^47^ Among those, six pointed out changes: the influence of new national syllabus guidelines in the more humanized practice of dentists, reinforcing the bond, the extended look at the territory and the community; and the potential work of community health care agent in the establishment of bonding, welcoming, and autonomy of users.^5^ ^,^ ^47^

We identified 11 studies^5^ ^,^ ^14^ ^,^ ^19^ ^,^ ^21^ ^,^ ^22^ ^,^ ^30^ ^,^ ^40^ ^,^ ^42^ ^,^ ^46^ ^,^ ^47^ ^,^ ^56^ out of the 12 that mentioned difficulties regarding the working conditions of SUS. Some critical bottlenecks were poor labor relationships, with small wages and unstable employment,^5^ ^,^ ^1^ ^9^ ^,^ ^22^ with probable effects on turnover and professional satisfaction, which jeopardizes the quality of health care;^5^ ^,^ ^22^ DS having double shifts in public and private health care units; and the lack of compliance with the weekly workload of 40 hours in FHCT, as something agreed to by managers and workers.^42^ ^,^ ^44^ The lack of financial, structural, physical, and human resources also influenced the working conditions.^5^ ^,^ ^30^

Among the 15 studies on permanent education in health, eight^18^ ^,^ ^19^ ^,^ ^21^ ^,^ ^22^ ^,^ ^26^ ^,^ ^30^ ^,^ ^39^ ^,^ ^44^ showed that professionals working in FHCT were not trained before starting their position,^44^ and that no training processes focusing on professional oral health training was available, to make care more complete.^18^ ^,^ ^19^ ^,^ ^26^ ^,^ ^30^ ^,^ ^38^ However, seven studies^10^ ^,^ ^13^ ^,^ ^16^ ^,^ ^29^ ^,^ ^34^ ^,^ ^39^ ^,^ ^42^ ^,^ ^45^ pointed changes: over 90.0% of the DS from a study^5^ reported taking part in training courses *–* the ones who did not had just been hired; another study^22^ showed that 67.8% of the DS had been trained to FHCT and felt the need to specialize in public health care, to be able to work in FHCT.

## DISCUSSION

Most of the analyzed studies were published from 2008, which indicates a recent interest in the field. The increased number of qualitative studies over the last few years adds an important dimension to the evaluation of actions in oral health, by producing knowledge from the experiences of professionals.

There was a predominance of studies from the Northeast region, which indicates that the results characterize a region, and not Brazil as a whole. According to Soares et al,^54^ as the Northeast region is the one with the highest number of family health care teams in the country, that might explain the predominance of studies in the region.

The most investigated work-analyzing elements in oral health in PHC were completeness, extension, and improvement of care. The literature suggests these are two of the most commonly analyzed elements, which may positively contribute to improving and orienting public policies (in overcoming inequalities in the access to health care services, in reaching equity in the system, and in achieving completeness regarding practices and teamwork).^5^ ^,^ ^52^

Despite the significant extension of coverage in oral health in PHC over the last decade, there are barriers that keep Brazilians from accessing the services.^24^ The advances in the extension and improvement of care to the population are few and the work process in oral health care may be damaged by the permanent excess demand and predominance of healing actions.

Among the principles and guidelines of SUS, completeness may be the least visible one in the path of the health care system and its practices.^24^ The study pointed out that completeness is insufficient, with weak points to be dealt with the work of FHCT teams. These analyses may be associated with the polysemy and coverage of the concept of completeness. To be effective, they require extended clinic, integration of individual and collective practices, and ability to solve problems with ensured access and articulation with other levels of care.^24^ ^,^ ^52^

Teamwork directly influences completeness.^58^ For oral health care professionals, integration in the work of family health care teams is limited by the historical isolation of these two professional categories, associated with the late introduction of oral health in FHCT and to the individualist and technicist training of professionals. That jeopardizes the full integration of the human being.^5^ ^,^ ^45^

Professionals will be required to learn and re-learn through their individual experiences in collective work situations. Work is the result of a debate over rules and values of a worker with themselves, about how to be able to manage the complexity of issues regarding collective work.^e^ In that sense, permanent education in health arises as a fundamental device. However, the review of the literature showed that training is focused on qualifying,^18^ which is generally distant from work routines and restricted to professional centers.

The intersectorial actions and fostering of popular participation and social control were not investigated to a great extent, which corroborates the literature.^54^ ^,^ ^55^ The volunteer nature of some teams or professionals may be partially explained by the fact that acting pursuant to the intersectoriality principle requires availability for periods that are not established in employment contracts.^15^ Despite the existence of a consensus regarding the need for intersectoriality in PHC, it is a process being built in FHCT.^48^ One of the challenges to implement it is the training of professionals, which is guided according to the perception of complexity of problems, and to the recognition of the need for intersectorial actions to intervene in such problems.^51^

The planning, monitoring, and evaluation of actions are far from the everyday routines of oral health care teams.^54^ It is a challenge that requires mobilization, engagement, and decision by managers and professionals.

Improving working conditions in SUS is directly related to improving the quality of care, but that is not a linear relationship. In contexts that are considered favorable to work, according to the principles of FHCT, teams focus their practices on treating occasional or scheduled patients.^25^ At the same time, in adverse contexts, professionals seek alternatives to be efficient.^6^ It is hard to define to which extent working conditions influence the change in professional practices.

The literature shows that, after a decade since the “Smiling Brazil” Program was implemented, the main problems and difficulties in the work of oral health care teams are not exclusive to dentistry. Strictly speaking, they follow the reality of FHCT teams, pursuant to what has been established by recent studies on the work of family health care teams.^2^ ^,^ ^49^ ^,^ ^56^

The advances are concentrated in educational and permanent education actions, in welcoming, bonding, and accountability. The main challenges are related to completeness; extension and improvement of care; integrated teamwork; working conditions; planning, monitoring, and evaluation of actions; stimulating people’s participation and social control; and intersectorial actions.

Despite the new regulatory environment, there are very few changes in oral health work. Professionals tend to reproduce the dominant biomedical model. Continuing efforts will be required in the management of work, training, and permanent education. Increasing the engagement of managers and professionals in the process to understand the dynamics of work and training in the perspective of building significant changes for local realities is one of the possibilities to enable the substitution of traditional practices and a new way to provide health care services.

## Footnotes

a Ministério da Saúde. Portaria nº 613, de 13 de junho de 1989. Aprova a Política Nacional de Saúde Bucal. *Diario Oficial Uniao*. 13 jun 1989; Seção I: 42607.

b Ministério da Saúde. Portaria nº 648, de 28 de março de 2006. Aprova a Política Nacional de Atenção Básica, estabelecendo a revisão de diretrizes e normas para a organização da Atenção Básica para o Programa Saúde da Família (FHCP) e o Programa Agentes Comunitários de Saúde (PACS). *Diario Oficial Uniao*. 29 mar 2006; Seção I: 71.

c Ministério da Saúde. Portaria nº 2.488, de 21 de outubro de 2011. Aprova a Política Nacional de Atenção Básica, estabelecendo a revisão de diretrizes e normas para a organização da Atenção Básica, para a Estratégia Saúde da Família (FHCT) e o Programa de Agentes Comunitários de Saúde (PACS). *Diario Oficial Uniao*. 24 out 2011; Seção I: 48.

d Ministério da Saúde, Secretaria de Atenção à Saúde, Departamento de Atenção Básica, Coordenação Nacional de Saúde Bucal. Diretrizes da Política Nacional de Saúde Bucal. Brasília (DF); 2004. Available from: http://dab.saude.gov.br/portaldab/biblioteca.php?conteudo=publicacoes/pnsb

e Schwartz Y. Trabalho e uso de si. *Pro-Posições*. 2000;1(5)(32):34-50, jul. 2000. Available from: http://www.proposicoes.fe.unicamp.br/proposicoes/textos/32-artigos-schwartzy.pdf
