# Supplementary material for: Advances and challenges in oral health after a decade of the “Smiling Brazil” Program
Source: Rev Saude Publica. 2016 Jan 14;49:98. doi: 10.1590/S0034-8910.2015049005961 (PMC4760711; doi:10.1590/S0034-8910.2015049005961)
Supplement: Translation

# Translation: Avanços e desafios da saúde bucal após uma década de Programa Brasil Sorridente

**Authors:** Charleni Inês Scherer, Magda Duarte dos Anjos Scherer

**Affiliations:** I Programa de Pós-Graduação em Saúde Coletiva. Faculdade de Ciências da Saúde. Universidade de Brasília. Brasília, DF, Brasil; II Departamento de Saúde Coletiva. Faculdade de Ciências da Saúde. Universidade de Brasília. Brasília, DF, Brasil

**Keywords:** Serviços de Saúde Bucal, organização e administração, Odontologia em Saúde Pública, recursos humanos, Atenção Primária à Saúde, Condutas na Prática dos Dentistas, Condições de Trabalho, Políticas Públicas de Saúde, Revisão

## Abstract

**OBJETIVO:**

Analisar as mudanças no trabalho em saúde bucal na atenção primária à saúde após o lançamento das Diretrizes da Política Nacional de Saúde Bucal.

**MÉTODOS:**

Foi realizada revisão da literatura nas bases de dados Medline, Lilacs, Embase, SciELO, Biblioteca Virtual em Saúde e *The Cochrane Library*, de 2000 a 2013, sobre elementos analisadores das mudanças no trabalho. Os descritores utilizados foram: atenção primária à saúde, saúde da família, trabalho, política de saúde, serviços de saúde bucal, odontologia, saúde bucal e Brasil. Foram selecionados e analisados 32 estudos, com predomínio de estudos qualitativos, da região Nordeste, com trabalhadores, sobretudo dentistas, e com foco na integralidade e qualificação da assistência.

**RESULTADOS:**

Os avanços observados concentraram-se nas ações educativas e de educação permanente; no acolhimento, vínculo e responsabilização. Os principais desafios estiveram relacionados à: integralidade; ampliação e qualificação da assistência; trabalho integrado em equipe; condições de trabalho; planejamento, monitoramento e avaliação das ações; estímulo à participação popular e ao controle social; e ações intersetoriais.

**CONCLUSÕES:**

Apesar do novo cenário normativo, as mudanças são incipientes no trabalho em saúde bucal. Os profissionais tendem a reproduzir o modelo biomédico dominante. Serão necessários esforços continuados no campo da gestão do trabalho, da formação e da educação permanente. Dentre as possibilidades, está a ampliação do engajamento dos gestores e dos profissionais num processo de compreensão da dinâmica do trabalho e da formação na perspectiva de construir mudanças significativas para as realidades locais.

## INTRODUÇÃO

O Brasil avançou com o Sistema Único de Saúde (SUS) ao estabelecer a universalidade e integralidade como princípios e a ampliação da cobertura da Atenção Primária à Saúde (APS), por meio da Estratégia Saúde da Família (ESF). Entretanto, prevalece o modelo biomédico assistencial à saúde, que orienta as práticas profissionais, inclusive da odontologia.^4^ ^,^ ^13^ ^,^ ^35^ ^,^ ^48^ ^,^ ^50^ ^,^ ^54^ ^,^ ^55^

Segundo o último levantamento epidemiológico nacional, o Brasil saiu da condição mundial de média para baixa prevalência de cárie.^59^ Embora os resultados sejam satisfatórios nacionalmente, chamam a atenção: (a) diferenças regionais na prevalência e gravidade da cárie são marcantes, o que indica necessidade de políticas voltadas para a equidade na atenção; (b) pouca redução da cárie na dentição decídua (18,0%) e 80,0% dos dentes afetados continuam não tratados; (c) o déficit em idosos é significativo, apesar das necessidades de próteses terem diminuído em adolescentes e adultos; e (d) prevalência de oclusopatia que requer tratamento em 10,0% dos adolescentes, o que sugere a necessidade de redimensionar a oferta de procedimentos ortodônticos na atenção secundária.^32^ ^,^ ^33^ Esses resultados estão associados ao perfil da prática odontológica, caracterizado pela realização de ações eminentemente clínicas, com ênfase em atividades restauradoras, e ações preventivas direcionadas a escolares, que se mostraram insuficientes para responder às necessidades da população.^31^

A trajetória brasileira de reorientação do modelo de atenção à saúde bucal na APS é marcada cronologicamente por quatro momentos, com potencial de impulsionar mudanças no trabalho: (i) primeira Conferência Nacional de Saúde Bucal (CNSB) em 1986, seguida da criação da Política Nacional de Saúde Bucal em 1989,^a^ e da segunda CNSB em 1993;^9^ 2º(ii) inserção dos profissionais de odontologia à ESF em 2000,^17^ enfrentando a restrição histórica de atuação junto ao grupo materno-infantil, e do estabelecimento de incentivo financeiro federal; criação das novas diretrizes curriculares nacionais dos cursos de graduação da área da saúde;^3^ e aprovação das normas e diretrizes de inclusão das equipes de saúde bucal na ESF em 2001; (iii) lançamento do Programa Brasil Sorridente em 2004^23^ e da terceira CNSB, que contribuiu para a produção democrática e progressista sobre a temática; (iv) responsabilidades e atribuições comuns e específicas dos profissionais de saúde bucal inseridas na Política Nacional de Atenção Básica em 2006^b^ e reiteradas em 2011.^c^

A trajetória recente na atenção à saúde bucal sinaliza um novo modelo em construção no País (Figura 1).

**Figura 1:** Linha do tempo, identificando os momentos e normatizações que marcam o incentivo às mudanças no processo de trabalho em saúde bucal na Atenção Primária à Saúde no Brasil.

CNSB: Conferência Nacional de Saúde Bucal; PSF: Programa Saúde da Família; ESF: Estratégia Saúde da Família; ESB: Equipe de Saúde Bucal

O Programa Brasil Sorridente, em formato de Diretrizes da Política Nacional de Saúde Bucal (PNSB),^d^ é o maior programa público de saúde bucal do mundo e completou uma década em 2014.^28^ Alterações foram provocadas no trabalho das equipes de saúde bucal na APS nesse período, de forma a cumprir com os objetivos de reorientação do modelo de atenção.^20^

A APS constitui-se de um espaço potencial de inovação na gestão e organização do processo de trabalho, um dos eixos centrais da reordenação da atenção à saúde no SUS.

O presente artigo teve por objetivo analisar as mudanças no trabalho em saúde bucal na atenção primária à saúde após lançamento das Diretrizes da Política Nacional de Saúde Bucal.

## MÉTODOS

A revisão da literatura foi guiada por elementos analisadores das mudanças no trabalho da saúde bucal na APS, segundo as normatizações vigentes^27^ ^,^ ^c^ ^,^ ^d^ e a partir da publicação das Diretrizes da PNSB. Foram utilizados os seguintes elementos de análise das mudanças em saúde bucal na APS: acolhimento, vínculo e responsabilização; ampliação e qualificação da assistência; ações intersetoriais; ações educativas; educação permanente; estímulo à participação popular e ao controle social; integralidade; planejamento, monitoramento e avaliação das ações; trabalho integrado em equipe; e condições de trabalho. As questões norteadoras foram: “Quais elementos analisadores são abordados?”, “Há relatos de mudanças, avanços ou dificuldades no trabalho?”, “Há recomendações, críticas ou sugestões para o trabalho em saúde bucal na APS?”.

Os estudos foram coletados nas bases Medline (via PubMed), Lilacs, Embase, SciELO, Biblioteca Virtual em Saúde e *The Cochrane Library* (via Bireme). Foram incluídas publicações a partir de 2000 a 2013 (até 3 de novembro de 2013). Foi incluído período anterior a 2004, quando lançadas as Diretrizes da PNSB, momento precedido de experiências e pactuações entre os diversos atores envolvidos na temática e que poderiam ter sido objeto de pesquisas publicadas em periódicos científicos.

Os descritores utilizados na busca na PubMed foram: (“*primary health care*” OR *“family health*” OR “*work*” OR *“health policy*”) AND (“*dental health services*” OR “*dentistry”* OR “*oral health*”) AND (“*Brazil*”). Os descritores foram utilizados em inglês e português nas demais bases. A combinação foi realizada com a utilização dos operadores booleanos “*AND*” e “*OR*” e utilizando termos do *Medical Subject Headings* (MeSH) ou análogos disponíveis em cada base pesquisada.

A coleta de dados foi realizada por uma das autoras, enquanto a seleção e avaliação dos estudos foi pareada por outras duas autoras familiarizadas com o tema e com o método. Foram encontrados 77 artigos na Medline, 538 na Lilacs, quatro na Embase*,* 67 no SciELO*,* 338 na BVS APS e 13 no *The Cochrane Library*, totalizando 1.037 artigos.

Foram incluídos os artigos que se referiam ao trabalho ou às práticas ou mudança de modelo na saúde bucal na APS, selecionados pelo título. Foram selecionados aqueles que problematizavam a organização e a gestão dos processos de trabalho em saúde bucal, em especial do trabalho em equipe na APS, um eixo central da reordenação da atenção à saúde no SUS. Foram excluídos os estudos de reflexão teórica, dissertações e teses.

Foram selecionados 211 artigos, dos quais foram excluídos 60 que se repetiram nas distintas bases, resultando em 151 artigos para leitura dos resumos e 52 para leitura integral. Ao final, 32 artigos foram incluídos na análise final (Figura 2).

**Figura 2:** Busca, seleção, inclusão e exclusão dos estudos sobre o processo de trabalho na saúde bucal na Atenção Primária à Saúde. APS: Atenção Primária à Saúde

Foram identificados aspectos gerais da publicação, características metodológicas e principais resultados, analisados a partir dos elementos (Tabela 1). A triagem das informações foi realizada independentemente pelos pesquisadores e comparada em reunião de consenso. Os itens consensuais foram considerados adequados e incluídos na descrição dos resultados. Estes foram agrupados em função das categorias temáticas prévias (elementos de análise) e analisados de forma descritiva.

**Tabela 1:** Aspectos gerais dos estudos selecionados, características metodológicas e sujeitos do estudo.

Ano	Local	Autoria	Revista	Título	Tipo de estudo e metodologia		Sujeitos do estudo
2013	SP	Pezzato LM, L’Abbate S e Botazzo C	Ciência & Saúde Coletiva, 18(7):2095-104	A produção de micropolíticas no processo de trabalho em saúde bucal: uma abordagem sócio-analítica	Qualitativo	Abordagem sócio analítica	Trabalhadores (ESB) e usuários
2013	MG	Sanglard-Oliveira CA, Werneck MAF, Lucas SD e Abreu MHNG	Ciência & Saúde Coletiva, 18(8):2453-60	Atribuições dos Técnicos em Saúde Bucal na Estratégia Saúde da Família em Minas Gerais, Brasil	Qualitativo	Estudo transversal e descritivo Entrevista telefônica	Trabalhadores (CD)
2013	PI	Moura MS, et al	Ciência & Saúde Coletiva, 18(2):471-80	Saúde bucal na estratégia de saúde da família em um colegiado gestor regional do estado do Piauí	Qualitativo	Estudo transversal Questionário	Trabalhadores (CD)
2012	ES	Esposti CDD, Oliveira AE, Santos Neto ET e Zandonade E	Saúde Soc. São Paulo, 21(2):372-85	O processo de trabalho do técnico em saúde bucal e suas relações com a equipe de saúde bucal na Região Metropolitana da Grande Vitória, Espírito Santo, Brasil	Qualitativo	Entrevista semiestruturada	Trabalhadores (CD)
2012	PE	Pimentel FC, et al	Cad. Saúde Pública, 28 Sup:S146-S157	Caracterização do processo de trabalho das equipes de saúde bucal em municípios do Estado de Pernambuco, Brasil, de acordo com o tamanho da população: a partir de links da comunidade para a organização dos cuidados clínicos.	Qualitativo	Questionário estruturado	Trabalhadores (ESB)
2011	SP	Cunha BAT, Marques RAA, Castro CGJ e Narvai PC	Saúde Soc, 20(4):1033-45	Saúde bucal em Diadema: da odontologia escolar à estratégia saúde da família	Qualitativo	Entrevista semiestruturada e análise documental	Gestores e trabalhadores (Prefeitos, SMS, CSB e CD)
2011	SP	Mialhe FL, Lefèvre F, Lefèvre AMC	Ciência & Saúde Coletiva, 16(11):4425-32	O agente comunitário de saúde e suas práticas educativas em saúde bucal: uma avaliação qualiquantitativa	Quali-Quanti	Entrevista semiestruturada	Trabalhadores (ACS)
2011	PE	Silva SF et al	Ciência & Saúde Coletiva, 16(1):211-20	Análise do avanço das equipes de saúde bucal inseridas na Estratégia Saúde da Família em Pernambuco, região Nordeste, Brasil, 2002 a 2005	Quantitativo	Estudo descritivo exploratório	Dados secundários (SIA-SUS)
2011	BA	Rodrigues AAAO, Nascimento MAA, Fonsêca GS e Siqueira DVS	Rev. Baiana Saúde Pública, 35(3):695-709	Saúde bucal na estratégia saúde da família em um município do semiárido baiano	Qualitativo	Entrevista semiestruturada e observação	Trabalhadores (ESF e ESB)
2010	PE	Pimentel FC, et al	Ciência & Saúde Coletiva, 15(4):2189-96	Análise da atenção à saúde bucal na Estratégia de Saúde da Família do Distrito Sanitário VI, Recife (PE)	Qualitativo	Estudo descritivo Entrevista semiestruturada e dados secundários	Gestores e trabalhadores (CSB, CD e enfermeira)
2010	BA	Rodrigues AAAO, Santos AM e Assis MMA	Ciência & Saúde Coletiva, 15(3):907-15	Agente comunitário de saúde: sujeito da prática em saúde bucal em Alagoinhas, Bahia	Qualitativo	Entrevista semiestruturada, observação e análise documental	Trabalhadores (ACS e ESB)
2010	CE	Nuto SAS, Oliveira GC, Andrade, JV e Maia MCG	Rev. APS, 13(4):505-9	O acolhimento em saúde bucal na estratégia de saúde da família, Fortaleza - CE: um relato de experiência	Qualitativo	Relato de experiência	Trabalhadores (ESB e ACS), usuários e acadêmicos
2010	PI	Moura MS, et al	Ciência & Saúde Coletiva, 15(supl.1): 1487-95	Perfil e práticas de saúde bucal do agente comunitário de saúde em municípios piauienses de pequeno porte	Qualitativo	Estudo transversal observacional descritivo Questionário	Trabalhadores (ACS)
2010	PE	Martelli PJL, et al	Ciência & Saúde Coletiva, 15(Supl. 2):3243-8	Perfil do cirurgião dentista inserido na Estratégia de Saúde da Família em municípios do estado de Pernambuco, Brasil	Qualitativo	Estudo descritivo de caso exploratório e transversal Questionário	Trabalhadores (CD)
2010	SC	Faccin D, Sebold R e Carcereri DL	Ciência & Saúde Coletiva, 15(Supl. 1):1643-52	Processo de trabalho em saúde bucal: em busca de diferentes olhares para compreender e transformar a realidade	Qualitativo	Entrevista semiestruturada	Trabalhadores (ESF e ESB)
2009	MG	Lourenço EC, Silva ACB, Meneghin MC e Pereira AC	Ciência & Saúde Coletiva, 14(Supl. 1):1367-77	A inserção dos serviços de saúde bucal no Programa Saúde da Família no Estado de Minas Gerais	Quali-Quanti	Questionários	Trabalhadores (ESF e ESB)
2009	PR/SP	Nascimento AC, Moysés ST, Bisinelli JC e Moysés SJ	Rev. Saúde Pública, 43(3):455-62	*Oral health in the family health strategy: a change of practices or semantics diversionism*	Qualitativo	Grupo Focal	Trabalhadores (CD)
2009	BA	Rodrigues AAAO, Gallotti AP, Pena SFA e Ledo CAS	Rev. Baiana Saúde Pública, 33(4):582-94	Saúde bucal no programa de saúde da família na cidade de Feira de Santana (BA): o perfil do cirurgião-dentista	Quantitativo	Questionário	Trabalhadores (CD)
2009	RN	Rocha ECA e Araújo MAD	Rev. Adm. Pública, 43(2): 481-517	Condições de trabalho das equipes de saúde bucal no Programa Saúde da Família: o caso do Distrito Sanitário Norte em Natal, RN	Quali-Quanti	Estudo exploratório descritivo Questionário	Trabalhadores (CD)
2009	RN	Holanda ALF, Barbosa AAA e Brito EWG	Ciência & Saúde Coletiva, 14(Supl. 1):1507-12	Reflexões acerca da atuação do agente comunitário de saúde nas ações de saúde bucal	Qualitativo	Relato de experiência	Trabalhadores (CD)
2008	PR	Koyashiki GAK, Alves-Souza RA e Garanhani ML	Ciência & Saúde Coletiva, 13(4):1343-54	O trabalho em saúde bucal do Agente Comunitário de Saúde em Unidades de Saúde da Família	Qualitativo	Entrevista	Trabalhadores (ACS)
2008	BA	Dos Santos AM, Assis MMA, Nascimento, MAA e Jorge, MSB	Rev. Saúde Pública 2008;42(3):464-70	Vínculo e autonomia na prática de saúde bucal no Programa Saúde da Família	Qualitativo	Abordagem crítico-reflexiva Entrevista semiestruturada e observação	Trabalhadores (ESF e ESB) e usuários
2008	BA	Chaves SCL e Vieira-da-Silva LM	*Health Policy* 86(1):119-28	*Inequalities in oral health practices and social space: an exploratory qualitative study*	Qualitativo	Estudo exploratório Entrevista semiestruturada	Trabalhadores e gestores (CD e CSB) e usuários
2008	PE	Martelli PJL, et al	Ciência & Saúde Coletiva, 13(5):1669-74	Análise do modelo de atenção à saúde bucal em municípios do estado de Pernambuco	Qualitativo	Entrevista semiestruturada	Gestores (CSB)
2008	PE	Pimentel FC, et al	Rev. Baiana Saúde Pública, 32(2): 253-64	Evolução da assistência em saúde bucal na estratégia de saúde da família do município do Recife (PE) no período de 2001 a 2007	Quantitativo	Estudo descritivo	Dados secundários (SIA-SUS)
2008	RN	Almeida GC e Ferreira MA	Cad. Saúde Pública, 24(9):2131-40	Saúde bucal no contexto do Programa de Saúde da Família: práticas de prevenção orientadas a saúde individual e pública	Quali-Quanti	Estudo exploratório descritivo Entrevista estruturada Análise documental	Trabalhadores (CD) Dados secundários (SIAB e SIA-SUS)
2008	PA	Emmi DT e Barroso RFF	Ciência & Saúde Coletiva, 13(1):35-41	Avaliação das ações de saúde bucal no Programa Saúde da Família no distrito de Mosqueiro, Pará	Qualitativo	Estudo descritivo Questionário	Usuários
2007	BA	Dos Santos AM, et al	Cad. Saúde Pública, 23(1):75-85	Linhas de tensões no processo de acolhimento das equipes de saúde bucal do Programa Saúde da Família: o caso de Alagoinhas, Bahia, Brasil	Qualitativo	Entrevista semiestruturada e observação	Trabalhadores (ESF e ESB) e usuários
2007	BA	Chaves SCL e Silva LMV	Ciência & Saúde Coletiva, 12(6):1697-710	As práticas profissionais no campo público de atenção à saúde bucal: o caso de dois municípios da Bahia	Qualitativo	Entrevista semiestruturada	Trabalhadores (CD)
2007	RN	Souza TM e Roncalli AG	Cad. Saúde Pública, 23(11):2727-39	Saúde bucal no Programa Saúde da Família: uma avaliação do modelo assistencial	Qualitativo	Entrevista estruturada, observação e análise documental	Gestores (CSB ou responsável) e trabalhadores (CD)
2005	PR	Baldani MH, Fadel CB, Possamai T e Queiroz MGS	Cad. Saúde Pública, 21(4):1026-35	Inclusão de serviços de saúde bucal no Programa Saúde da Família no Estado do Paraná	Quali-Quanti	Questionários	Gestores (CSB ou SMS) e trabalhadores (CD)
2005	BA	Rodrigues AAAO e Assis MMA	Rev. Baiana Saúde Pública, 29(2):273-85	Oferta e demanda na atenção à saúde bucal: o processo de trabalho no Programa Saúde da Família em Alagoinhas – Bahia	Qualitativo	Entrevista semiestruturada, observação e análise documental	Gestores (Prefeito, SMS e CSB) e trabalhadores (ESF e ESB + ACS)

ACS: Agente Comunitário de Saúde; CD: Cirurgião Dentista; CSB: Coordenador de Saúde Bucal; ESB: Equipe de Saúde Bucal; ESF: Equipe de Saúde da Família; SIA-SUS: Sistema de Informações Ambulatoriais do SUS; SIAB: Sistema de Informação de Atenção Básica; SMS: Secretário Municipal de Saúde

## RESULTADOS

A maioria das pesquisas era da região Nordeste (n = 21; 65,6%), Sudeste (n = 6; 18,7%) e Sul (n = 3; 9,3%) (Tabela 1). Foram identificados estudos qualitativos (n = 24; 75,0%) e quantitativos (n = 3; 9,3%). Em 15,6% dos artigos, utilizou-se combinação de métodos quanti e qualitativos (Tabela 2). Em geral, os principais participantes foram trabalhadores da ESF, destacando o cirurgião-dentista (CD). Cerca de 1/3 incluíram gestores e usuários (Tabela 1). Todos os estudos (n = 32) abordaram um ou mais elementos de análise do trabalho, e nenhum contemplou todos eles (Tabela 2).

**Tabela 2:** Estudos selecionados que abordam os elementos analisadores do trabalho em saúde bucal na Atenção Primária à Saúde no Brasil apontando desafios e mudanças.

Região	UF	Ano	Autoria	Elementos analisadores presentes nos estudos	

				Indica(m) desafio(s)	Aponta(m) mudança(s)
Sul	PR	2005	Baldani MH, Fadel CB, Possamai T e Queiroz MGS	1, 7, 9 e 10	4
	PR	2008	Koyashiki GAK, Alves-Souza RA e Garanhani ML	5 e 9	7
	SC	2010	Faccin D, Sebold R e Carcereri DL	1, 3, 7, 9 e 10	2
Sudeste	MG	2009	Lourenço EC, Silva ACB, Meneghin MC e Pereira AC	5, 8, 9 e 10	1
	SP	2010	Cunha BAT, Marques RAA, Castro CGJ e Narvai PC	1 e 7	4 e 5
	SP	2011	Mialhe FL, Lefèvre F, Lefèvre AMC	2 e 5	0
	ES	2012	Esposti CDD, Oliveira AE, Santos Neto ET e Zandonade E	9	5 e 10
	SP	2013	Pezzato LM, L’Abbate S e Botazzo C	6	1 e 7
	MG	2013	Sanglard-Oliveira CA, Werneck MAF, Lucas SD e Abreu MHNG	0	2, 4 e 7
Sul e Sudeste	PR	2009	Nascimento AC, Moysés ST, Bisinelli JC e Moysés SJ	1, 9	4, 7
	SP			1	0
Nordeste	BA	2005	Rodrigues AAAO e Assis MMA	7 e 9	0
	BA	2007	Santos AM, Assis MMA, Rodrigues AAAO, Nascimento MAA, Jorge MSB	4	0
	BA	2007	Chaves SCL e Silva LMV	7	0
	RN	2007	Souza TM e Roncalli AG	1, 8, 9 e 10	3
	BA	2008	Santos AM, Assis MMA, Nascimento MAA e Jorge MSB	1 e 10	4
	BA	2008	Chaves SCL e Vieira-da-Silva LM	2 e 7	0
	PE	2008	Martelli PJL, et al	1, 5, 8 e 10	9
	PE	2008	Pimentel FC, et al	7 e 8	1
	RN	2008	Almeida GC e Ferreira MA	1 e 8	2
	BA	2009	Rodrigues AAAO, Gallotti AP, Pena SFA e Ledo CAS	10	2 e 5
	RN	2009	Rocha ECA e Araújo MAD	1 e 10	0
	RN	2009	Holanda ALF, Barbosa AAA e Brito EWG	7 e 9	5
	PE	2010	Pimentel FC, et al	1, 6, 7, 8, 9	2
	BA	2010	Rodrigues AAAO, Santos AM e Assis MMA	9	7
	CE	2010	Nuto SAS, Oliveira GC, Andrade JV e Maia MCG	1	4 e 5
	PI	2010	Moura MS, et al	7	5
	PE	2010	Martelli PJL, et al	1, 5 e 10	2
	PE	2011	Silva SF, et al	7	1 e 5
	BA	2011	Rodrigues AAAO, Nascimento MAA, Fonsêca GS e Siqueira DVS	5, 7, 8 e 10	0
	PE	2012	Pimentel FC, et al	1, 5, 6, 7 e 8	9
	PI	2013	Moura MS, et al	5, 7, 9 e 10	0
Norte	PA	2008	Emmi DT e Barroso RFF	0	1 e 2

1: ampliação e qualificação da assistência; 2: ações educativas; 3: ações intersetoriais; 4: acolhimento, vínculo e responsabilização; 5: educação permanente em saúde; 6: estímulo à participação popular e ao controle social; 7: integralidade; 8: planejamento, monitoramento e avaliação das ações; 9: trabalho integrado em equipe; 10: condições de trabalho; 0: Ausência de elementos analisadores no estudo; UF: Unidade da Federação; PR: Paraná; SC: Santa Catarina; MG: Minas Gerais; SP: São Paulo; ES: Espírito Santo; BA: Bahia; PE: Pernambuco; RN: Rio Grande do Norte; PI: Piauí; PA: Pará

Os avanços incipientes no trabalho em saúde bucal situaram-se em: ações educativas; ações de educação permanente; acolhimento, vínculo e responsabilização. As principais dificuldades e desafios estão relacionados à: integralidade; ampliação e qualificação da assistência; trabalho em equipe; planejamento, monitoramento e avaliação das ações; e condições de trabalho. Poucos estudos^14^ ^,^ ^36^ ^,^ ^38^ ^,^ ^39^ ^,^ ^57^ abordaram o estímulo à participação popular e ao controle social e ações intersetoriais.

Dos 32 estudos analisados, 19 abordavam o tema integralidade, dos quais 14^5^ ^,^ ^7^ ^,^ ^8^,^10^ ^,^ ^14^ ^,^ ^16^ ^,^ ^29^ ^,^ ^30^ ^,^ ^37^ ^,^ ^38^ ^,^ ^41^ ^,^ ^44^ ^,^ ^53^ apontaram dificuldades para reestruturar a saúde bucal na APS, superar as práticas do modelo tradicional da odontologia escolar e criar novas possibilidades, como a abordagem familiar e o diagnóstico da situação de saúde.^1^ ^,^ ^10^ ^,^ ^14^ ^,^ ^34^ ^,^ ^39^ Persistiram ações com foco no atendimento clínico e valorização excessiva da técnica e da especialidade e houve predomínio de práticas preventivas e educativas tradicionais.^7^ ^,^ ^14^ ^,^ ^41^ As equipes de saúde bucal encontraram dificuldades para as práticas pertinentes à ESF, como visitas domiciliares pelo dentista, ações de prevenção de agravos e de promoção à saúde, bem como reuniões e ações para a articulação com a comunidade.^5^ ^,^ ^39^

Mudanças incipientes vinculadas à integralidade foram apresentadas em cinco estudos^18^ ^,^ ^32^ ^,^ ^36^ ^,^ ^43^ ^,^ ^45^ (26,3%), que relataram a introdução do atendimento centrado no usuário com espaço para o diálogo e para o encontro de saberes instituintes da saúde bucal. Ao contrário das atividades individuais, as ações em grupos e os avanços na visão preventiva e na prática de educação em saúde dos profissionais foram expressivas na ESF.^1^ ^,^ ^14^ ^,^ ^38^ Outra mudança refere-se aos técnicos em saúde bucal, que desprendiam seu tempo mais em atividades preventivas e coletivas do que em atividades assistenciais.^45^

Dois estudos abordaram o trabalho em equipe e mostraram que a maioria dos CD relatou integração com a equipe, porém poucos participavam das reuniões e utilizavam prontuários únicos.^5^ ^,^ ^19^ O trabalho do CD raramente se inseria em práticas partilhadas com profissionais de outras áreas, uma vez que suas ações eram desenvolvidas de forma autônoma, independente e individualizada.^39^

Houve entraves para o trabalho em equipe também entre os profissionais da odontologia.^13^ Os CD reconheciam que a relação com os técnicos em saúde bucal era prejudicada pela desinformação sobre o processo de trabalho, pela falta de clareza sobre a responsabilidade legal dos CD em relação às atividades dos técnicos, bem como pelo receio quanto à possibilidade de os técnicos tornarem-se dentistas práticos e tomarem seu espaço no mercado de trabalho. Por outro lado, os CD valorizavam a participação dos técnicos na reorganização do trabalho odontológico e na construção de uma relação de parceria e cooperação.

As ações intersetoriaisforam abordadas em dois dos 32 estudos,^14^ ^,^ ^57^ relacionadas às ações de prevenção e educação em saúde bucal desenvolvidas na comunidade ou nas escolas.^57^ A intersetorialidade apresentou-se como prática voluntária em parte das equipes e não conduzia aos impactos esperados. Tal circunstância pode levar os profissionais ao descrédito em relação à ESF, considerando a incapacidade do setor saúde enfrentar isoladamente os determinantes sociais do processo saúde-doença.

Ações educativasestiveram presentes em nove estudos,^1^ ^,^ ^8^ ^,^ ^11^ ^,^ ^14^ ^,^ ^22^ ^,^ ^26^ ^,^ ^38^ ^,^ ^42^ ^,^ ^45^ dos quais sete apontaram avanços nas práticas dos profissionais em relação ao preconizado pelas diretrizes da PNSB. Segundo Martelli et al,^22^ 92,3% dos CD as consideraram pertinentes e, desses, 89,6% relataram realizá-las. Rodrigues et al^42^ identificaram que todos os CD realizavam atividades de educação em saúde, na unidade de saúde ou em escolas, asilos, igrejas e creches. As ações mais relatadas foram as de prevenção e promoção em grupos,^1^ ^,^ ^14^ com destaque para os técnicos em saúde bucal como responsável por elas.^45^ As de educação em saúde estiveram mais presentes no cotidiano dos profissionais da ESF.^38^ ^,^ ^42^

Segundo Mialhe et al,^26^ as atividades educativas em saúde bucal eram realizadas esporadicamente e voltadas prioritariamente às gestantes, mães e crianças, em um modelo vertical de transmissão de informações, visando mudanças de comportamentos individuais e incorporação de hábitos saudáveis. Essa visão era compartilhada pela população, que considerava as orientações de higiene bucal como uma das melhorias mais importantes.^11^

Oito estudos^1^,^19^ ^,^ ^21^ ^,^ ^37^ ^-^ ^39^ ^,^ ^44^ ^,^ ^57^ relataram que o planejamento, o monitoramento e a avaliação das ações eram práticas incipientes e indicaram dificuldades na realização de levantamentos para reconhecer as necessidades da população, considerando as características sociais e epidemiológicas.^38^ ^,^ ^44^ Apesar dos avanços, a saúde bucal necessita de muito investimento, além do controle e avaliação de suas ações por meio dos sistemas de informação, fortalecendo o planejamento e a tomada de decisão.^37^

O estímulo à participação popular e ao controle social foi abordado em três estudos^36^ ^,^ ^38^ ^,^ ^39^ como uma ação a ser fomentada pelas equipes. Pezatto et al^36^ ressaltaram que a apropriação dos temas da saúde bucal pelos espaços do controle social é um dos desafios para a implementação dos serviços de saúde bucal no SUS.

A ampliação e qualificação da assistênciaem saúde bucal foram abordadas em 19 dos 32 estudos. Desses, cinco^11^ ^,^ ^19^ ^,^ ^36^ ^,^ ^37^ ^,^ ^53^ abordaram avanços, porém as dificuldades prevaleceram (73,6%) em relação ao atendimento às demandas da população adscrita.^5^ A demanda excessiva destacou-se como ponto negativo, com predominância das ações curativas pelo CD.^19^ Mesmo com a ampliação do acesso aos serviços de saúde bucal, a organização da demanda foi um nó crítico, pois há várias portas de entrada, grande demanda reprimida e pouca oferta.^19^ ^,^ ^32^ ^,^ ^34^ Ampliar e qualificar a assistência requer enfrentar desafios relacionados ao investimento público insuficiente; às dificuldades de encaminhamento para especialidades; às ações com foco no atendimento clínico com valorização excessiva da técnica e da especialidade; e à crescente demanda por serviços pela população, focalizadas nas ações curativas.^34^ Apesar das dificuldades, pontos positivos foram observados nos estudos: delimitação da clientela, possibilitando melhor acompanhamento; mudança no perfil dos procedimentos odontológicos realizados; e cobertura populacional de acordo com o mínimo estipulado pelo Ministério da Saúde.^11^ ^,^ ^37^ ^,^ ^53^

Estabelecer o acolhimento, vínculo e responsabilização permite a negociação entre usuários e profissionais do cuidado integral à saúde, contribuindo para que o ato terapêutico esteja centrado no profissional, mas que seja realizado pela manifestação de desejo do usuário. Esses elementos foram abordados por sete estudos.^5^ ^,^ ^10^ ^,^ ^32^ ^,^ ^34^ ^,^ ^45^ ^-^ ^47^ Desses, seis apontaram mudanças: a influência das novas diretrizes curriculares nacionais na prática mais humanizada de odontólogos, reforçando o vínculo, o olhar ampliado para o território e para a comunidade; e o potencial trabalho dos agentes comunitários em saúde no estabelecimento de vínculo, acolhimento e autonomia dos usuários.^5^ ^,^ ^47^

Foram identificados 11 estudos^5^ ^,^ ^14^ ^,^ ^19^ ^,^ ^21^ ^,^ ^22^ ^,^ ^30^ ^,^ ^40^ ^,^ ^42^ ^,^ ^46^ ^,^ ^47^ ^,^ ^56^ dos 12 que abordavam dificuldades relativas às condições de trabalho no SUS. Foram nós críticos: a precarização das relações de trabalho com baixos salários e contratações instáveis,^5^ ^,^ ^19^ ^,^ ^22^ com prováveis reflexos na rotatividade e na satisfação profissional, comprometendo a qualidade da assistência à saúde;^5^ ^,^ ^22^ a dupla jornada de trabalho do CD entre o setor público e o privado; e o descumprimento da jornada semanal de 40 horas na ESF como algo pactuado entre gestores e trabalhadores.^42^ ^,^ ^44^ A falta de recursos financeiros, estruturais, físicos e humanos também refletiu nas condições de trabalho.^5^ ^,^ ^30^

De 15 estudos que abordavam a educação permanente em saúde, oito^18^ ^,^ ^19^ ^,^ ^21^ ^,^ ^22^ ^,^ ^26^ ^,^ ^30^ ^,^ ^39^ ^,^ ^44^ identificaram que profissionais que atuavam na ESF eram capacitados após inserção na estratégia, sem preparo prévio^44^ e que não havia processos formadores voltados à qualificação profissional em saúde bucal, de modo a tornar a prática do cuidado mais integral.^18^ ^,^ ^19^ ^,^ ^26^ ^,^ ^30^ ^,^ ^38^ Entretanto, sete estudos^10^ ^,^ ^13^ ^,^ ^16^ ^,^ ^29^ ^,^ ^34^ ^,^ ^39^ ^,^ ^42^ ^,^ ^45^ apontaram mudanças: mais de 90,0% dos CD de um estudo^5^ afirmaram ter participado de cursos de capacitação e os que não participaram tinham sido recém-contratados; outro estudo^22^ mostrou que 67,8% dos CD possuíam formação voltada para a ESF e sentiam necessidade de fazer especialização em saúde pública, buscando a aptidão necessária para o trabalho na ESF.

## DISCUSSÃO

A maioria dos estudos analisados foi publicada a partir de 2008, o que indica recente interesse na área. O aumento de estudos qualitativos nos últimos anos acrescenta uma dimensão importante à avaliação das ações na área da saúde bucal ao produzir conhecimento a partir da experiência dos profissionais.

Predominaram estudos da região Nordeste, indicando que os resultados caracterizam uma região e não o País em sua totalidade. Segundo Soares et al,^54^ como o Nordeste é a região com maior número de equipes de saúde da família no País, isso poderia explicar o predomínio de estudos na região.

Os elementos de análise do trabalho em saúde bucal na APS mais investigados foram a integralidade e a ampliação e qualificação da assistência. A literatura sugere que esses são dois dos elementos mais comumente analisados e que podem contribuir de maneira positiva para melhoria e orientação das políticas públicas (na superação de desigualdades no acesso aos serviços de saúde, no alcance da equidade dentro do sistema e na efetivação da integralidade relativa às práticas e ao trabalho em equipe).^5^ ^,^ ^52^

Apesar da significativa ampliação de cobertura de atenção à saúde bucal na APS na última década, existem barreiras de acesso que privam brasileiros dos serviços.^24^ São poucos os avanços na ampliação e qualificação da assistência à população e o processo de trabalho em saúde bucal pode ser prejudicado pela permanente demanda excessiva e predomínio de ações curativas.

Dentre os princípios e diretrizes do SUS, talvez o da integralidade seja o menos visível na trajetória do sistema de saúde e de suas práticas.^24^ O estudo apontou que a integralidade é incipiente e com fragilidades a serem enfrentadas no trabalho das equipes da ESF. Essas análises podem estar associadas à polissemia e à abrangência do conceito de integralidade. Para sua efetividade, são necessárias clínica ampliada, integração de práticas individuais e coletivas e resolutividade com garantia de acesso e articulação com outros níveis de atenção.^24^ ^,^ ^52^

O trabalho em equipe influencia diretamente a integralidade.^58^ Para os profissionais de saúde bucal, a integração no trabalho da equipe de saúde da família está limitada pelo histórico isolamento dessas categorias profissionais, associado à introdução tardia da saúde bucal na ESF e à formação individualista e tecnicista dos profissionais. Isso dificulta a abordagem integral do ser humano.^5^ ^,^ ^45^

Será preciso que os profissionais aprendam e reaprendam com a experiência vivida na singularidade das situações de trabalho coletivo. O trabalho é resultado de um debate de normas e de valores do trabalhador consigo mesmo, sobre como estar em condições de gerir a complexidade de questões relacionadas ao trabalho coletivo.^e^ Nesse sentido, a educação permanente em saúde surge como dispositivo fundamental. Porém, a revisão da literatura mostrou que a formação concentra-se em capacitações,^18^ em geral distantes do cotidiano de trabalho e restritas a núcleos profissionais.

As ações intersetoriais e estímulo à participação popular e ao controle social foram pouco investigados, coincidindo com a literatura.^54^ ^,^ ^55^ O caráter voluntário de algumas equipes ou profissionais pode ser parcialmente explicado pelo fato de que atuar segundo o princípio da intersetorialidade requer disponibilidade para períodos não estabelecidos no contrato de trabalho.^15^ Apesar de existir consenso acerca da necessidade da intersetorialidade na APS, ela é um processo em construção na ESF.^48^ Um dos desafios para que ela se efetive é a formação de profissionais orientada à percepção da complexidade dos problemas e ao reconhecimento da necessidade de ações intersetoriais para intervir em tais problemas.^51^

O planejamento, monitoramento e avaliação das ações estão distantes do cotidiano da equipe de saúde bucal.^54^ É um desafio que exige mobilização, engajamento e decisão de gestores e profissionais.

A melhoria das condições de trabalho no SUS está diretamente relacionada à melhoria da qualidade da assistência, mas não se trata de uma relação linear. Em contextos considerados favoráveis ao trabalho, segundo os princípios da ESF, as equipes centram suas práticas no atendimento à demanda espontânea ou programada.^25^ Ao mesmo tempo, em contextos adversos, profissionais buscam alternativas para serem eficazes.^6^ É difícil precisar em que medida as condições de trabalho influenciam a mudança das práticas profissionais.

A literatura mostra que, após uma década de implantação do Programa Brasil Sorridente, os principais problemas e dificuldades no trabalho das equipes de saúde bucal não são exclusivos da odontologia. A rigor, acompanham a realidade do trabalho das equipes da ESF, em consonância com o indicado por estudos recentes sobre o trabalho das equipes de saúde da família.^2^ ^,^ ^49^ ^,^ ^56^

Os avanços concentram-se nas ações educativas e de educação permanente, no acolhimento, vínculo e responsabilização. Os principais desafios estão relacionados à: integralidade; ampliação e qualificação da assistência; trabalho integrado em equipe; condições de trabalho; planejamento, monitoramento e avaliação das ações; estímulo à participação popular e ao controle social; e ações intersetoriais.

Apesar do novo cenário normativo, as mudanças são incipientes no trabalho em saúde bucal. Os profissionais tendem a reproduzir o modelo biomédico dominante. São necessários esforços continuados na gestão do trabalho, formação e educação permanente. Ampliar o engajamento dos gestores e dos profissionais no processo de compreensão da dinâmica do trabalho e da formação na perspectiva de construir mudanças significativas para as realidades locais é uma das possibilidades para que haja substituição das práticas tradicionais e um novo modo de se fazer saúde.

## Footnotes

a Ministério da Saúde. Portaria nº 613, de 13 de junho de 1989. Aprova a Política Nacional de Saúde Bucal. *Diario Oficial Uniao*. 13 jun 1989; Seção I: 42607.

b Ministério da Saúde. Portaria nº 648, de 28 de março de 2006. Aprova a Política Nacional de Atenção Básica, estabelecendo a revisão de diretrizes e normas para a organização da Atenção Básica para o Programa Saúde da Família (PSF) e o Programa Agentes Comunitários de Saúde (PACS). *Diario Oficial Uniao*. 29 mar 2006; Seção I: 71.

c Ministério da Saúde. Portaria nº 2.488, de 21 de outubro de 2011. Aprova a Política Nacional de Atenção Básica, estabelecendo a revisão de diretrizes e normas para a organização da Atenção Básica, para a Estratégia Saúde da Família (ESF) e o Programa de Agentes Comunitários de Saúde (PACS). *Diario Oficial Uniao*. 24 out 2011; Seção I: 48.

d Ministério da Saúde, Secretaria de Atenção à Saúde, Departamento de Atenção Básica, Coordenação Nacional de Saúde Bucal. Diretrizes da Política Nacional de Saúde Bucal. Brasília (DF); 2004. Disponível em: http://dab.saude.gov.br/portaldab/biblioteca.php?conteudo=publicacoes/pnsb

e Schwartz Y. Trabalho e uso de si. *Pro-Posições*. 2000;1(5)(32):34-50, jul. 2000. Disponível em: http://www.proposicoes.fe.unicamp.br/proposicoes/textos/32-artigos-schwartzy.pdf
